# Real-time intraoperative diagnosis by deep neural network driven multiphoton virtual histology

**DOI:** 10.1038/s41698-019-0104-3

**Published:** 2019-12-17

**Authors:** Sixian You, Yi Sun, Lin Yang, Jaena Park, Haohua Tu, Marina Marjanovic, Saurabh Sinha, Stephen A. Boppart

**Affiliations:** 1 Beckman Institute for Advanced Science and Technology, University of Illinois at Urbana-Champaign, Urbana, IL USA; 2 Department of Bioengineering, University of Illinois at Urbana-Champaign, Urbana, IL USA; 3 Department of Electrical and Computer Engineering, University of Illinois at Urbana-Champaign, Urbana, IL USA; 4 Department of Computer Science and Engineering, University of Notre Dame, Notre Dame, IN USA; 5 Carle Illinois College of Medicine, University of Illinois at Urbana-Champaign, Champaign, IL USA; 6 Departement of Computer Science, University of Illinois at Urbana-Champaign, Urbana, IL USA

**Keywords:** Cancer imaging, Cancer microenvironment

## Abstract

Recent advances in label-free virtual histology promise a new era for real-time molecular diagnosis in the operating room and during biopsy procedures. To take full advantage of the rich, multidimensional information provided by these technologies, reproducible and reliable computational tools that could facilitate the diagnosis are in great demand. In this study, we developed a deep-learning-based framework to recognize cancer versus normal human breast tissue from real-time label-free virtual histology images, with a tile-level AUC (area under receiver operating curve) of 95% and slide-level AUC of 100% on unseen samples. Furthermore, models trained on a high-quality laboratory-generated dataset can generalize to independent datasets acquired from a portable intraoperative version of the imaging technology with a physics-based adapted design. Classification activation maps and final feature visualization revealed discriminative patterns, such as tumor cells and tumor-associated vesicles, that are highly associated with cancer status. These results demonstrate that through the combination of real-time virtual histopathology and a deep-learning framework, accurate real-time diagnosis could be achieved in point-of-procedure clinical applications.

## Introduction

Histopathology is the gold standard for tissue assessment in clinical decision making and in research.^1^ However, conventional histological preparation and analysis is known to be time-consuming and labor intensive.^2,3^ Because of the need to use tissue fixation, paraffin embedding, sectioning, and histochemical staining, diagnosis is usually made after a considerable wait time (days), which prevents intraoperative assessment and delays treatment plans. While frozen sectioning can accelerate the histologic assessment of tissue during intraoperative procedures, the tens of minutes required, and the quality of the stained tissue sections, are often compromising.^4^ Therefore, imaging scientists have put extensive efforts into developing real-time histopathology and achieved significant advances.^5–9^ Label-free virtual histopathology is even more attractive for its real-time imaging capabilities utilizing intrinsic molecular contrast.^10–12^ Based on intrinsic structural, molecular, and metabolic contrast, label-free virtual histopathology visualizes tissue with comparable or even richer diagnostic value than conventional histopathology, and offers the unique potential to do such imaging in real-time, even in vivo.

These new imaging techniques, however, yield highly multidimensional datasets and involve new and unconventional biomolecular markers. Interpretation and classification of these images usually requires thorough visual inspection by an experienced biomedical engineer, biologist, or pathologist with extensive knowledge of the target domain. As with conventional histological images of hematoxylin and eosin (H&E)-stained tissue sections, interpretation by imaging scientists is labor-intensive, time-consuming, and susceptible to inter-observer variability.^13,14^ Efforts have been made to automate this process by feature engineering and multilayer perceptrons.^10^ Here, we propose to further advance this approach by utilizing recent advances in machine learning, specifically with deep neural nets (DNNs), that obviate the subjective and intellectually demanding feature extraction step, for real-time label-free virtual histopathology images.^11,12,15,16^

We recently developed a technique for slide-free virtual histochemistry based on stain-free slide-free multimodal multiphoton microscopy that simultaneously generates up to four intrinsic histochemical contrasts (two/three-photon autofluorescence for functional, molecular, and metabolic information, and second/third harmonic generation for structural information) from in vivo animal and ex vivo human tissue.^15^ In contrast to conventional histochemistry, our tetra-modal imaging shows great potential for fast general-purpose cell phenotyping because a relatively large number (four) of strictly spatially and temporally colocalized intrinsic contrasts can be imaged in tissue without interference. This allows visualization of a variety of cellular and stromal components in living or freshly excised tissue, including tumor cells, immune cells, endothelial cells, vasculature ducts, alveoli, cell niches, blood vessels, and collagen structures in the same composite image. The capability to obtain such rich molecular and structural information within seconds makes this technique an attractive alternative or adjunct to histochemistry in clinical settings, especially in time-sensitive scenarios such as the intraoperative assessment of breast tissue during surgical oncology procedures. To make real-time intraoperative diagnosis truly viable, computer-assisted analysis is needed to deliver prompt, reproducible, and accurate tissue assessment of these multidimensional virtual slides containing new biomarker information. No existing tool offers this automated analysis capability for the above-mentioned technology, but one can recognize the analogous application that recent and improving computer-aided diagnosis (CAD) image processing algorithms have had on identifying suspicious lesions in X-ray mammography images.^17^ The main technical challenge in developing such computational tools arises from the limited available datasets from the new technology. Furthermore, as with most new technologies, various degrees of discrepancies in image generation quality exist among different implementations of the technology. Therefore, an automated analysis method that can learn specific disease (cancer)-related features efficiently from limited datasets without losing the capability to generalize to other diseases or other imaging systems is greatly needed.

**Fig. 1:**
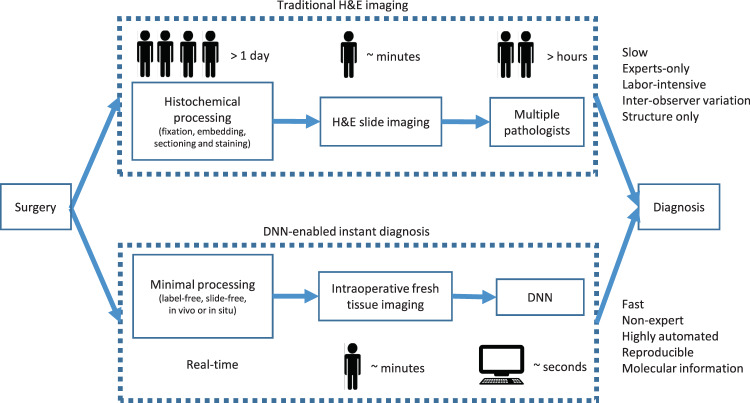
Proposed workflow for real-time intraoperative diagnosis during breast cancer surgeries. Instead of waiting days for histological and histochemical processing, we propose to image the tissue immediately after it is resected, or even directly in the cavity, and then analyze the images in real-time with a DNN-based classifier, thus generating results (probability of being cancerous) within minutes.

Recent years have witnessed a rapid surge in the development and demonstration of DNNs.^18–24^ Relying on a flexible combination of layers of convolutional masks, variations of DNNs have demonstrated their potential for diverse tasks and achieved unprecedented results for classifying, segmenting, and even synthesizing natural scene and medical images.^25,26^ Previous work on classifying traditional histopathology (H&E) images from non-small cell lung cancer using a DNN showed a superior accuracy of 97%.^14^ For breast cancer classification, an accuracy of 83.3% for differentiating between cancer and normal was achieved by combining convolutional neural networks based on H&E-stained breast biopsy images.^27^ With the rich molecular information afforded by our label-free technology,^11,12,15,16,28^ a well-designed DNN is expected to learn from the multimodal optical signatures and correlations and automate the analysis of virtual histology in real time (Fig. 1).

This study developed such a deep-learning framework for virtual histology to enable the workflow of real-time cancer diagnosis for (but not limited to) the intraoperative assessment of breast cancer. The framework was developed and tested using holdout-validation on a dataset generated by applying our previously demonstrated imaging system to fresh human breast tissue. To further validate our classifier, we tested it on independent datasets obtained from a portable intraoperative version of the system^16^ using the same imaging physics and contrast generating mechanisms. To improve the performance of domain adaptation, custom-designed data augmentation was introduced to the original laboratory-generated dataset to improve the generalizability of the DNN model. A physics-based design that reflects the optical and operating parameters of the portable system was used to model the quality degradation associated with the new test images, and to artificially degrade the original high-quality images, which were then used to retrain the classifier. This allowed the classifier to achieve a high level of test accuracy on images from the portable system without requiring an additional and separate training dataset representing the system (or more generally, other similar systems). Our study demonstrates that a DNN model combined with state-of-the-art multimodal multiphoton virtual histology provides a real-time, automated, and accurate method for real-time histology that can facilitate intraoperative assessment as well as pre- and post-operative diagnosis of biopsies and tissues for breast cancer.

## Results

### Deep-learning framework for classification

**Fig. 2:**
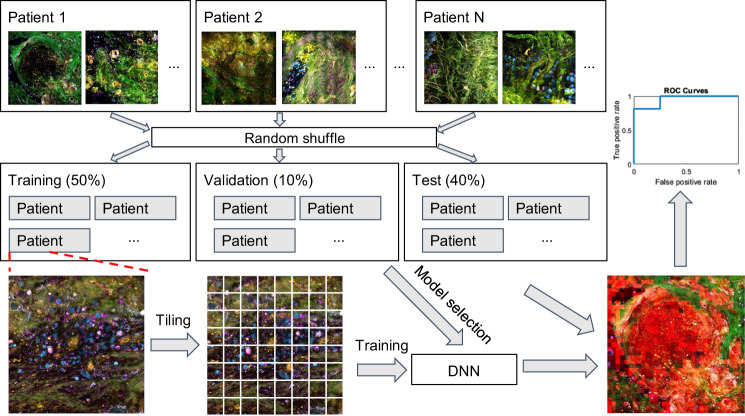
Deep-learning framework for training and evaluating a model to recognize cancer tissue from normal tissue. Subjects were randomly divided into three sets: training, validation, and test sets. Each subject was represented by multiple virtual slides, and each slide was sliced into smaller ‘tiles’. Model selection was done based on the performance in the validation set. After learning and selection, the model was applied to tiles in the previously unseen test data. This produces a heatmap of each slide showing the confidence of the model in each tile being cancerous. Per-tile accuracy was obtained from this heatmap (across all slides from test subjects) and per-slide accuracy was obtained by averaging tile-level predictions across the entire slide.

We sought to develop a deep-learning framework for automatic classification of real-time histopathology and test this model on independent data obtained using a portable intraoperative system. For the operating room-based data, we collected 99 images (a total of 25 Mpixels) from 22 cancer subjects and 31 images (a total of 8 Mpixels) from 7 normal subjects. For laboratory-based data, we collected 69 sets of mosaicked images (a total of 447 Mpixels) from 14 cancer subjects and 42 mosaicked images (a total of 323 Mpixels) from 7 normal subjects. Our task becomes feasible due to the heterogeneity of the tissue microenvironment within subjects, which allows for model-training from a large number of small ‘tiles’ representing different areas of each virtual slide (defined here as a large field-of-view image mosaicked from tiles from one imaging site), as well as the potentially dominant molecular changes between subjects.^16,29^ The challenge is to optimize the accuracy of the model without risking overfitting. This challenge was addressed by careful design of training and test datasets, as shown in Fig. 2, including testing on independent subjects and eventually on independent datasets from another cohort and another imaging system. To test the accuracy of our model, the data (122 virtual slides) were split into three sets based on random shuffling of the subjects within the same group (cancer and normal): training, validation, and testing (there was no subject overlap among these three sets). Considering the size of the input to the DNN as well as the number of training samples, random cropping was performed in the training process to generate batches of 256 × 256-pixel tiles from the original virtual slides, resulting in thousands of tiles. The training set that was fed into the DNN was comprised of a large collection of tiles from subjects in the training set, while excluding any data from subjects in the validation or test set. This method maximizes the size of the training set and avoids training and testing on tiles originating from the same human subjects, preventing the classifier from relying on intra-subject correlations between samples and resulting in inflated estimates of accuracy.

### Accurate classification of virtual histology

**Fig. 3:**
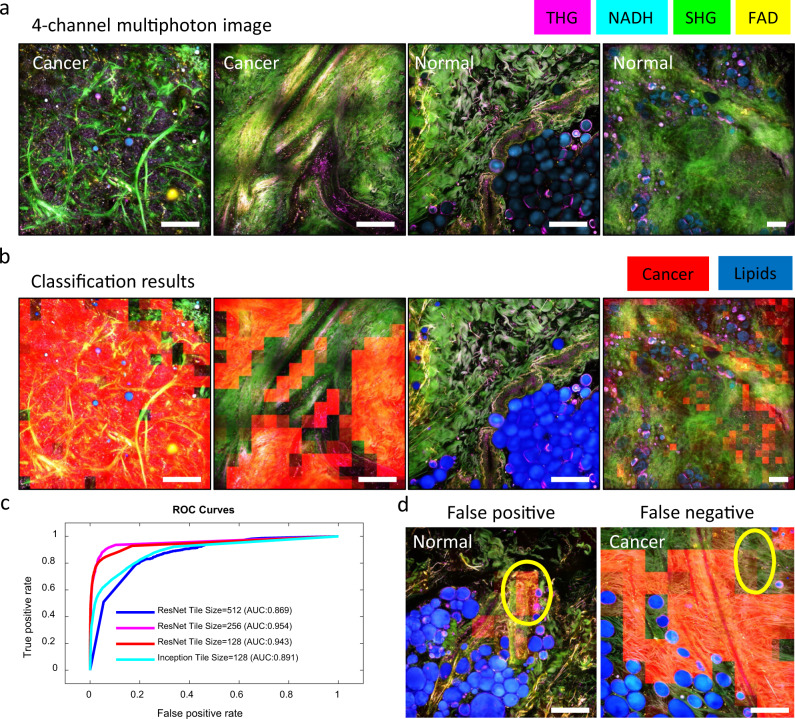
Classification of human breast cancer by a DNN. **a** Multiphoton composite image of the four channels (THG, NADH, SHG, FAD) for two cancer and two normal slides. **b** Classification results corresponding to the multiphoton images in **a**. The cancer probability map was coded with red color and overlaid with the original multiphoton composite image. To avoid the bias of low-impact high-frequency content, lipid-filled adipocytes were segmented out (blue mask) as a preprocessing step prior to classification. **c** AUC statistics on test set, for different model architectures and different tile sizes. **d** Representative example of false positive (normal tiles classified as cancer, highlighted by the yellow oval) and false negative (cancer tiles classified as normal, highlighted by the yellow oval). Scale bar: 200 µm.

Based on the framework presented in Fig. 2, a DNN was first trained to classify cancer from normal tissue using laboratory-based virtual histology images produced by our previously published technique.^11,12,15^ The DNN predicts a cancer versus normal probability score for each tile, allowing us to create a heatmap that highlights regions likely to be cancerous in each image (Fig 3a, b). It is to be noted that a significant portion of breast tissue is adipocytes for both cancer and normal subjects. As interpretation of images relies heavily on distinguishing between tumor, tumor stroma, and normal stroma, label clean-up before classification is warranted. Thus, we pre-processed all images by automatic segmentation and excluded adipocytes by using a popular semantic segmentation neural network, U-Net,^20^ to reduce the bias generated by adipocytes and to enable efficient identification of more relevant cancer-associated image content. All evaluations reported below were obtained based on pre-processed training, validation, and test sets containing tiles that were not dominated by adipocytes (<50% of the area within each tile).

**Fig. 4:**
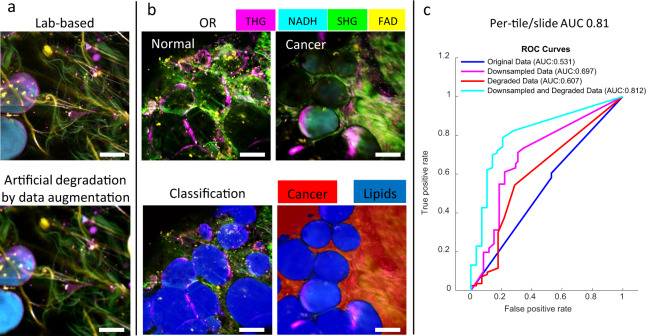
Testing on an independent intraoperative dataset. **a** Original laboratory-based image (top) and its artificially degraded version (bottom) that reflects image quality characteristics learnt from the parameters and settings of the intraoperative system. **b** Upper panel shows multiphoton composite images of the four channels (THG, NADH, SHG, FAD) acquired by the portable system for sample images from a normal cancer-free subject and a cancer subject, and the lower panel shows corresponding classification results. The cancer probability map was coded with a red color and overlaid with the original multiphoton composite image. Similar to Fig. 3, lipids were first segmented out (blue mask) as a preprocessing step prior to classification. **c** Accuracy statistics (AUC) on an intraoperative dataset when model was trained with original laboratory-based data (blue), with downsampled original laboratory-based data (purple), with physics-based augmented data minus the downsampling (red), or with artificially degraded (downsampling and degradation) laboratory-based data (cyan). Scale bar: 50 µm.

The accuracy was assessed both at the tile and slide level. Similar to previous work,^10,14^ the per-slide accuracy was obtained by averaging the probabilities for all the tiles within the slide. Optimization of the network architecture as well as the tile size was guided by per-tile statistics (AUC, area under curve). As shown in Fig. 3c, a ResNet-20-based DNN architecture^19^ with an input tile size of 256 × 256 pixels generates the highest test AUC (95.2% per tile, 100% per slide). The comparison among different tile sizes reflects the trade-offs between the number of available “independent” tiles (smaller sizes provide more tiles) and the field-of-view (greater tile size allows the model to capture more global information) in each training session. The superiority of the ResNet model with tile sizes of 128 × 128 or 256 × 256 compared to 512 × 512 reflects that the model benefits from a larger training set while still capturing sufficient cancer-distinguishing features in a tile. Interestingly, as shown in Fig. 3c, on the per-tile level, a false positive prediction is highly associated with normal ducts and vessels in normal tissue, while a false negative call is usually found in cancer tissues that are mostly collagen fibers with no visible abnormalities (angiogenesis, vesicles, etc). On the per-slide level, however, these errors are eliminated by aggregating the predicted scores of all the tiles within the slide, which showed the importance of the “big picture” view of the classifier—a comprehensive view of the microenvironment in addition to the micron-level details within each tile. It appears that the main reason for the false sensitivity is the intrinsic scarcity of vessel- and duct- structures in the normal tissue, as well as the limited field-of-view of the environmental context. There are two potential approaches that can further improve the algorithm. First, combining local environmental details and a more global context in the training process will help the network learn both the greater perspective as well as the details that are correlated with cancer diagnosis. This can be achieved by incorporating a downsampled image of the entire slide (context) with the original multi-channel tiles (local details) if given more independent subject numbers in a future study. Second, including more normal ducts and vessels in the normal training dataset will reduce the false positive rate of the algorithm. This modification will penalize the direct association of ducts and vessels with cancer and avoid the bias introduced by the imbalance of data complexity between the cancer group and the normal group (Fig. 4).

### Accurate classification of independent intraoperative data demonstrates generalizability of the DNN model

We next subjected the above methodology to a stronger test of utility by evaluating the classifier on independent datasets collected from a recently developed portable system that can be deployed in more demanding operating room settings.^16^ This intraoperative dataset shares the same imaging mechanism and content (harmonic generation and autofluorescence) as the laboratory-based dataset used above. However, due to the engineering, design, and performance trade-offs that were needed to construct a compact and portable imaging system, image quality is lower than the laboratory-based system, with confounding factors such as bright ambient light in the operating room and limited acquisition time, compared to the laboratory-based datasets, which were acquired from a high-end, custom-built microscope^11,12,15^ in a totally dark, well-controlled setting. Due to these multiple trade-offs, direct application of the trained DNN from the previous section results in an AUC of 53%, only marginally better than a ‘coin toss’ call. This mediocre ability to adapt is in sharp contrast to human perception—human brains have little difficulty recognizing patterns in previously unseen lower-quality images after being ‘trained’ on high-quality data. We thus hypothesized that by adding human intuition of this quality difference into the model-training process, we can potentially improve domain adaptation without the model having ‘seen’ the new data.

We adopted a physics-based learning strategy to achieve our goal. First, considering the difference in system design and operation between the two datasets, data augmentation with the purpose of artificial degradation was introduced to the original high-quality laboratory-based image data by modelling difference in point spread function (PSF), floor vibrations, ambient light noise, and spatial sampling rate (see Methods). Most importantly, downsampling was used to reduce dependence on local features, and thus to improve robustness for real-life data, which resulted in an AUC of 69.7%. In addition, artificial degradation was added to the original laboratory-based data to mimic real-life data, which resulted in an AUC of 60.7%. Finally, a DNN model was trained on the augmented laboratory-based data (downsampling + degradation), and applied to the independent intraoperative dataset, achieving 81% AUC (per tile and per slide). Given that the new datasets were acquired using a totally different system and in an active clinically-driven intraoperative setting, these results are encouraging as they reflect the potential generalizability of the classification method, and the broader applicability of this approach to different diagnostic scenarios. The artificial degradation was based on a few well-established physical principles for characterizing image quality, rather than an elaborate highly parameterized model, thereby arguing for its feasibility in future cases of domain adaptation.

### Interpretation of the DNN classification model

**Fig. 5:**
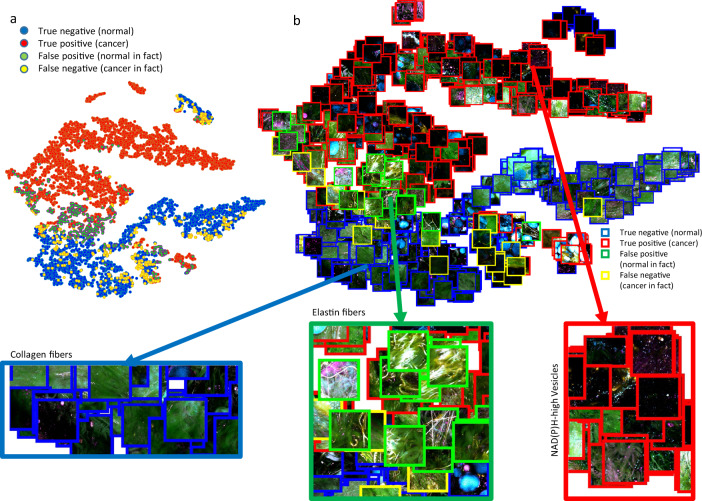
t-SNE representation of the penultimate-layer features used by the DNN. **a** Dot-represented plot was generated by t-SNE projection of the feature vector of the penultimate layer of all the tiles being classified. **b** Tile-embedded plot was generated by substituting a subset of the dots (randomly sampled) in **a** with their original multiphoton image.

In search of an intuitive understanding of the image features used by the trained DNN, we first extracted for each sample tile the neuron activity profile in the penultimate layer of the network. This 512-dimensional vector acts as input to the final neuron that makes the decision to classify the image as cancer or normal, and may thus be considered as a compact representation of the image that captures its salient features for discerning its class. We further compressed these representative vectors of images into a two-dimensional feature space using t-SNE,^30^ and created a scatter plot where each image is placed at the location determined by its two-dimensional t-SNE projection. This allows us to visualize the collection of images on a ‘canvas’ where images are clustered by their mutual similarity as defined by the DNN. We can see in the resulting plot (Fig. 5) that the DNN tends to cluster tiles with similar optical signatures and shapes. Tiles of green collagen fibers (lower left), yellow elastin fibers (center left), and blue vesicles (upper right) are located in separate clusters. For the t-SNE plot presented in Fig. 5, traditional Euclidean distance with perplexity 30 was used. The t-SNE representations used in this paper demonstrated reasonable stability when testing with different parameters for optimization and datapoints, as shown in Supplementary Fig. 1 and Supplementary Notes.

**Fig. 6:**
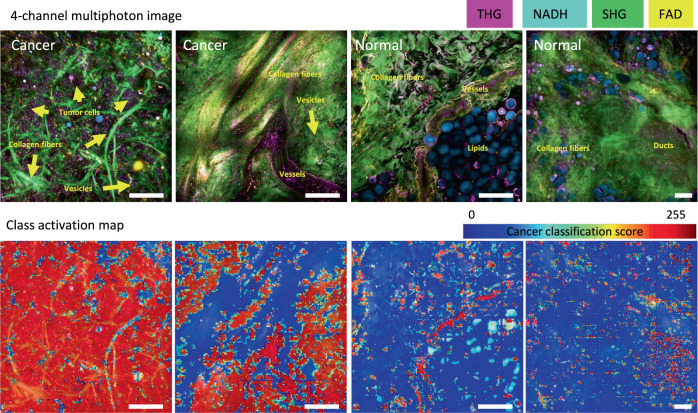
The representative class activation maps of cancer and normal tissue. The maps highlight the discriminative image regions used for cancer classification, e.g., tumor cells, vessels, ducts, and vesicles. Scale bar: 200 µm.s.

To better understand which pixels and spatial patterns influence the final decisions by the DNN, we used a discriminative localization technique called Classification Activation Mapping (CAM) that provides insights into the regions of interest in this classification task.^31^ CAM highlights the regions in the original image that contribute to a positive (cancer) class prediction by the DNN, thus drawing our attention to discriminative regions in each image. We observe (Fig. 6) that the high activation regions frequently correspond to objects indicative of clusters of tumor cells, vesicles, vessels, and ducts, which is in concordance with our previous observations.^29^ In contrast, collagen fibers and lipids are seen to have much lower association with cancer, which is understandable due to their extensive presence in both normal and cancer tissues. This visualization helped ensure that the trained models, with their numerous parameters, capture interpretable features that are reasonable in light of prior knowledge of cancer biology, and also helped discover potential patterns for cancer characterization. More work needs to be done in the future to further explore the features and patterns used by this and other DNNs.

## Discussion

This study developed a deep-learning framework for label-free virtual histology, with the ultimate goal of enabling real-time intraoperative diagnosis of breast cancer. We demonstrate that such a prediction framework can accurately discriminate cancer samples from normal ones, even when trained with modest sized datasets. We are particularly encouraged by the high accuracy in differentiating Stage I invasive ductal carcinoma from normal human breast (97% accuracy), which can be challenging for conventional diagnosis based on H&E (reported under-interpretation of 13% and an overall concordance rate of 75.3% among pathologists).^32^ An important challenge overcome was that of significant difference in image quality between laboratory-acquired image data and intraoperatively-acquired image data, with the latter obtained from a different, portable version that still utilized the same underlying nonlinear optical physics and imaging technology. We addressed this challenge of domain adaptation to intraoperative images by introducing physics-based intuition into the model-training phase, without extra cost of new data, similar to recent reports on the benefit of physics-based learned models.^33^ In addition to cancer/normal classification, precise cancer staging is of important clinical interest due to its impact on prognosis and treatment. With larger datasets in the near future, the presented framework can be easily adapted to the staging task as the DNN can be exposed to more of the diversity and the heterogeneity correlated with specific cancer stages.

Our study demonstrates that a DNN model combined with a state-of-the-art stain-free slide-free virtual histology platform may provide a real-time, automated, and accurate method for real-time histology that can facilitate intraoperative assessment. However, we also would like to point out several practical considerations for clinical adoption of this approach. First, as demonstrated by other clinical studies using nonlinear excitation, laser safety needs to be rigorously investigated.^34^ We have performed continuous hour-long imaging on living animals without observing laser-induced damage.^15^ More systematic analysis of potential laser-induced damage needs to be performed for human tissue before final adoption. Second, the cost of this imaging system is indeed higher than conventional microscopes ($80k vs $8k). However, the high cost of this current setup is mostly due to the requirement of a femtosecond laser, which is highly likely to drop in cost due to the prospering industry of femtosecond fiber lasers.^35^ Finally, additional operational readiness requires active engagement of all the key stakeholders, which is challenging, given the history of 20 years of development and adoption of digital pathology.^36,37^ Despite these challenges, the adoption and use of this technology is promising, due to the rapid growth, integration, and impact of the machine learning and medical engineering communities.^38,39^

## Methods

### Virtual histology image datasets

Laboratory-based virtual histology images were collected by a custom-built benchtop multimodal multiphoton microscope from fresh human breast tissue (normal: 7 subjects, 42 virtual slides; cancer: 12 subjects, 69 virtual slides).^11,12^ Independent intraoperative images were collected by a portable imaging system from resected fresh human breast tissue in the operating room (normal: 7 subjects, 31 virtual slides; cancer: 22 subjects, 99 virtual slides).^16^ This study was conducted in accordance with a protocol approved by the Institutional Review Boards at the University of Illinois at Urbana-Champaign and Carle Foundation Hospital, Urbana, IL. All human tissue samples were obtained from subjects who preoperatively provided written informed consent permitting the investigational use of their tissue. Normal breast tissue samples were obtained from female subjects with no history of cancer who were undergoing breast reduction surgery. Cancerous breast tissue samples were obtained from female subjects diagnosed by a board-certified pathologist as having invasive ductal carcinoma who were undergoing mastectomy or lumpectomy procedures as part of their standard-of-care treatment for their disease. Histological slides and pathological diagnoses were obtained postoperatively for labels, which were used as our gold standard.

### Image preprocessing

To remove tiles dominated by adipocytes (low useful information), adipocytes were first segmented at the pixel level from the original images using U-Net.^20^ For this segmentation task, the training set contains raw 4-channel images and the manually annotated lipid mask (identified by comparing to conventional histology).

### Training the DNN

For experiments related to Fig. 3, we used 50% of the data for training, 10% for validation, and 40% for final testing. This percentage allocation was selected to maximize the training sets while preserving a number of completely unseen subjects (randomly selected) for final testing. The input to the algorithm was the raw four-channel virtual slides (multiphoton images) together with the labels generated by the lipid segmentation network and the labels of being diagnosed as cancer or normal (generated by pathologist). All input images were converted to 8 bit representation and saved directly onto the GPU memory to facilitate access for the training process later. During each iteration, a mini-batch of four sets of 256 × 256 × 4 (or other tile size) tiles was randomly cropped from randomly chosen virtual slides for both cancer and normal groups on the condition that the chosen tile was not dominated by adipocytes (determined by the input lipid segmentation mask). The loss function was defined as the cross entropy between the ground truth (labels by a pathologist) and the predicted probability. An Adam optimizer was used for iteration steps, with a learning rate of 0.0005, weight decay of 0.9, momentum of 0.999, and epsilon of 1 × 10^−8^. The DNNs included in this study were based on ResNet-20^19^ and Inception v3.^40^

### Adapting the model to new independent intraoperative datasets

To simulate the image artifacts and degradations associated with the intraoperative portable system and to apply them to the high-quality data, we first convolved the laboratory-based data with the measured PSF (with a full width half maximum 10% larger than the original system) of the portable system, which essentially blurred the images to a similar degree shared by the intraoperative data. Ambient light was modelled by additive white Gaussian noise for each individual channel based on prior knowledge of the measurement of the spectral response to the room light in the operating room.^41,42^ In addition, random shifting of pixels and lines was applied to the raw images to mimic floor vibrations in the operating room setting. Finally, to shift the attention away from local features and to more global features, as well as to compensate for the lower spatial sampling frequency of the intraoperative data, downsampling by a factor of four was applied to both laboratory-based and intraoperative data in the training and testing process. The final input tile was 128 × 128 pixels with the field-of-view of 512 × 512 μm^2^.

### Statistical analysis

The performance of the classifier was assessed using a testing set, which contained virtual slides from independent subjects that were never seen in the training or validation sets. The ROC curves and AUC were computed using Matlab.

## Supplementary information

Supplementary Material

Reporting Sum

## Data Availability

The data that support the findings of this study are available from the corresponding author (S.A.B.) upon reasonable request and through collaborative investigations.

## Code availability

The codes that are used to train, apply, and visualize the deep-learning model in this manuscript are available from the corresponding author (S.A.B.) upon reasonable request and through collaborative investigations.
