# Supplementary material for: Real-time intraoperative diagnosis by deep neural network driven multiphoton virtual histology
Source: NPJ Precis Oncol. 2019 Dec 17;3:33. doi: 10.1038/s41698-019-0104-3 (PMC6917773; doi:10.1038/s41698-019-0104-3)
Supplement: Supplementary file 1 — Supplementary Material [file 41698_2019_104_MOESM1_ESM.pdf]

Supplementary Materials

Supplementary notes

Stability test of t-SNE

The traditional Euclidean distance with perplexity 30 was used for t-SNE calculations in the original experiment shown in Fig. 5. Given the random initialization in the non-convex optimization process, it would be helpful to evaluate the stability of t-SNE plots in terms of variation of parameters in the optimization process as well as data points. Therefore, we tested the stability of the t-SNE plots by four experiments recommended by a t-SNE characterization paper[1], as demonstrated in the figure below.

Specifically, experiment 1 analyzes the geometric variations by introducing datapoints in different random orders. Experiment 2 analyzes the influence of the parameters in the resulting projections by using a wide set of recommended perplexity values ("may be viewed as a knob that sets the number of effective nearest neighbors")[2]. Experiment 3 analyzes the effect of number of datapoints in the resulting embeddings. Experiment 4 works with two randomly sampled (different random seeds) data points (4000 data points each) from the same distribution. We can see that there are changes in the absolute position of the datapoints in the resulting t-SNE projections, which is expected due to the randomness in initialization. However, what remains unchanged throughout these experiments is the clustering effect of cancer and normal group, which demonstrates the reasonable stability of using t-SNE projections for clustering/distinguishing features of cancer and normal images. We have added descriptions of the parameters and stability of t-SNE in the main text and added the above "experiments" to the supplementary materials and believe this will strength the credibility of the DNN-extracted features.

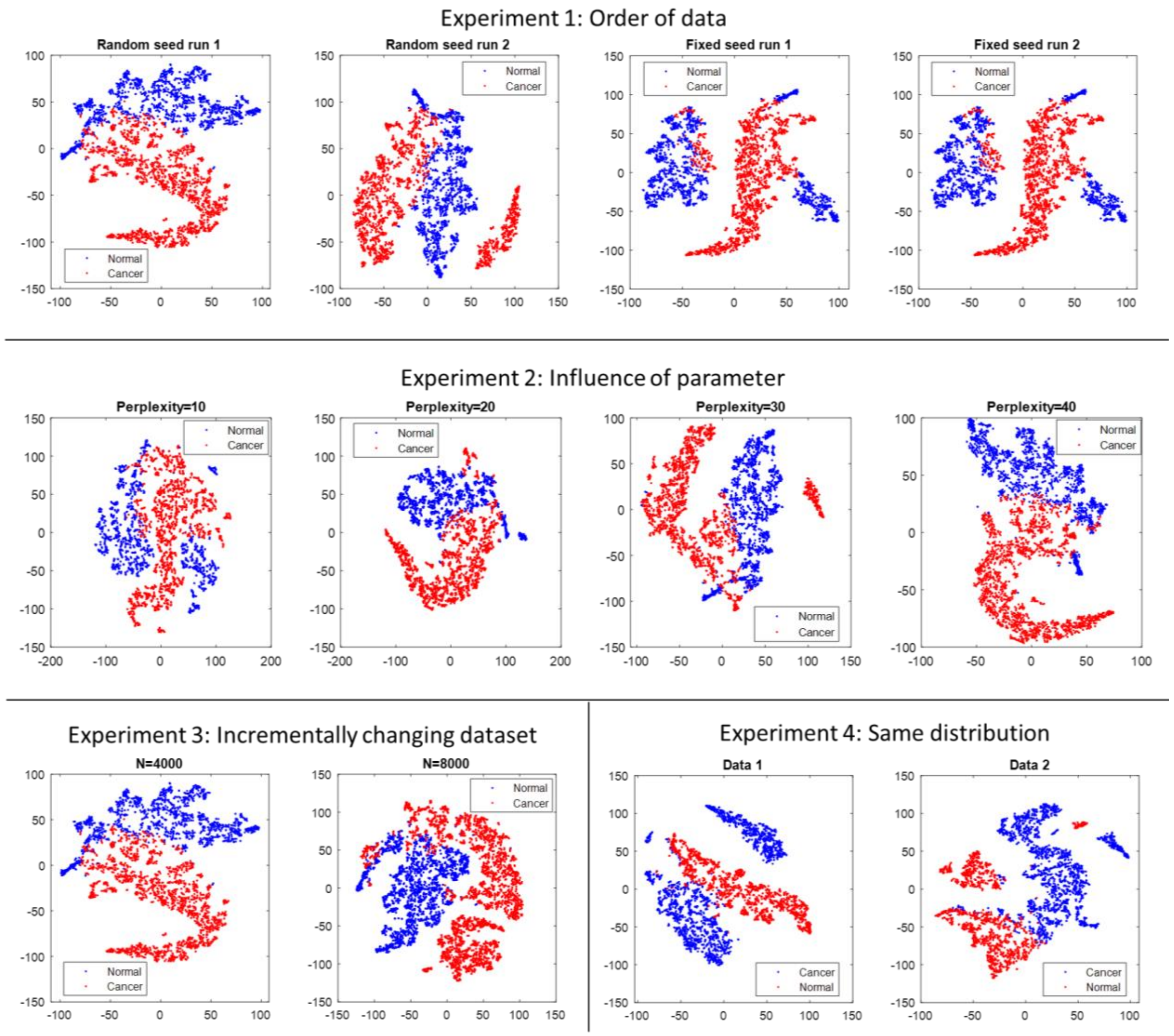

Supplementary Figure 1 Experimental results of testing t-SNE stability. Experiment 1 introduces 4000 data points in different random orders. Experiment 2 works with different perplexity values using identical datasets. Experiment 3 uses a dataset of 4000 points and 8000 points. Experiment 4 uses two different datasets (randomly sampled) from the same data distribution.

Supplementary References

1. García-fernández, F. J., Verleysen, M., Lee, J. A. & Díaz, I. Stability comparison of dimensionality reduction techniques attending to data and parameter variations. *Eurographics Conf. Vis.* (2013).

2. Matten, L. van der. Visualizing data using t-SNE. *J. Mach. Learn. Res.* **9**, 2579–2605 (2008).
